# Serum microRNAs as predictors of risk for non-muscle invasive bladder cancer

**DOI:** 10.18632/oncotarget.24473

**Published:** 2018-02-12

**Authors:** Jie Lian, Shu-Hong Lin, Yuanqing Ye, David W. Chang, Maosheng Huang, Colin P. Dinney, Xifeng Wu

**Affiliations:** ^1^ Department of Epidemiology, The University of Texas MD Anderson Cancer Center, Houston, TX 77030, USA; ^2^ Department of Urology, The University of Texas MD Anderson Cancer Center, Houston, TX 77030, USA

**Keywords:** serum miRNA, biomarker, expression ratio, risk score, non-muscle-invasive bladder cancer

## Abstract

MicroRNAs (miRNAs) are implicated in the development of nearly all cancers and may function as promising biomarkers for early detection, diagnosis and prognosis. We sought to investigate the role of serum miRNAs as potential diagnostic biomarkers or biomarkers of risk for early-stage bladder cancer. First, we profiled global serum miRNAs in a pilot set of 10 non-muscle invasive bladder cancer (NMIBC) cases and 10 healthy controls matched on age, gender and smoking status. Eighty nine stably detectable miRNAs were selected for further testing and quantification by high-throughput Taqman analysis using the Fluidigm BioMark HD System to assess their association with NMIBC risk in both discovery and validation sets totaling 280 cases and 278 controls. We found miR-409-3p and six miRNAs expression ratios were significantly associated with risk of bladder cancer in both discovery and validation sets. Interestingly, we identified expression of miR-409-3p and miR-342-3p inversely correlated with age and age of onset of NMIBC. A risk score was generated based on the combination of three miRNA ratios (miR-29a-3p/miR-222-3p, miR-150-5p/miR-331-3p, miR-409-3p/miR-423-5p). In dichotomized analysis, we found individuals with high risk score showed increased risk of bladder cancer in the discovery, validation, and combined sets. Pathway enrichment analyses suggested altered miRNAs and cognate target genes are linked to the retinoid acid receptor (RAR) signaling pathway. Overall, these results suggested specific serum miRNA signatures may serve as noninvasive predictors of NMIBC risk. Biological insights underlying bladder cancer development based on the pathway enrichment analysis may reveal novel therapeutic targets for personalized medicine.

## INTRODUCTION

Bladder cancer is the second most common genitourinary malignancy worldwide. In the United States, bladder cancer is the fourth most common cancer in men. The estimate for new cases is 76,960 and for deaths is 16,390 in 2016 [1]. Non-muscle invasive bladder cancer (NMIBC), including stage Ta and T1 and carcinoma *in situ* (CIS), accounts for 70%–80% of all newly diagnosed bladder cancer cases [2]. When bladder cancer is detected early, the 5-year survival rate is approximately 94% [3]. Therefore, it is important to identify patients at early stage for improved outcome. The current gold standard for diagnosis of bladder cancer remains white light cystoscopy of bladder coupled with urine cytology [4]. However, cystoscopy is an uncomfortable and costly invasive procedure, while the sensitivity of urine cytology for the detection of early-stage tumors is low as there are typically few cells found in urine [4]. Extensive research has attempted to identify early detection biomarkers, but these assays are either limited with high false positive rate or low sensitivity [5]. These limitations underscore the need for novel biomarkers, particularly noninvasive biomarkers in serum or plasma, for early detection or diagnosis of bladder cancer.

Over the last decade, numerous studies have demonstrated the potent pro- and anti-tumorigenic functions of microRNAs (miRNAs), which are a class of small noncoding RNAs that play a central role in the regulation of mRNA expression [6]. MiRNAs are frequently deregulated in bladder cancer and could contribute to bladder cancer development, progression and metastasis [7]. Additional studies have documented the existence of a large number of stable miRNAs in human blood samples with altered levels in cancer patients, which opened up the prospect of using circulating miRNAs as non-invasive cancer biomarkers [8, 9]. For example, serum miR-21 has been identified as a promising biomarker for the early detection and prognosis of colorectal cancer [10]. A miRNA panel consisting of 7 miRNAs provided a high diagnostic accuracy of hepatocellular carcinoma [11] and a five circulating miRNA signature has been identified for the diagnosis of very high-risk prostate cancer [12]. Although a recent study of serum miRNA expression from genome-wide profiling has been conducted for NMIBC [13], the study population involved East Asians and similar studies on Caucasians are not found.

In the present multiphase study, we first performed pilot screening to determine the expression profiles of 754 serum miRNAs using Taqman miRNA arrays in patients with NMIBC and healthy controls from hospital and clinic-derived non-Hispanic Caucasian population. Next, stably detectable miRNAs were selected and simultaneously quantified in two additional discovery and validation sets by high-throughput, multiplex quantitative real-time reverse-transcription PCR (qRT-PCR) analysis in order to evaluate the clinical significance of these miRNAs as potential biomarkers for diagnosis and risk prediction of bladder cancer. In addition, predicted targets of the miRNAs were analyzed *in silico* to identify enrichment in pathways, yielding possible underlying biological mechanisms for future experimental verification.

## RESULTS

### Patient characteristics

The patient characteristics for 140 NMIBC patients and 139 healthy controls in each discovery and validation set were summarized in Table 1. There were no significant differences in the distribution of age and sex between the discovery and validation sets between cases and controls. A statistically significant higher proportion of ever smokers was observed in cases compared to controls (*P* < 0.001).

**Table 1:** Host characteristics of patients with NMIBC and controls

Variables		Discovery set			Validation set		
		Cases *N* = 140, *N* (%)	Controls *N* = 139, *N* (%)	*P*-value^*^	Cases *N* = 140, *N* (%)	Controls *N* = 139, *N* (%)	*P-*value^*^
Age (y)							
Mean (SD)		64.91 (10.87)	65.14 (10.17)	0.860	64.36 (11.04)	64.29 (9.92)	0.952
Pack year^**^							
Mean (SD)	38.76 (29.48)		26.71 (22.51)	**0.005**	38.48 (31.02)	33.52 (29.66)	0.292
Sex							
Male	127 (90.71)		127 (91.37)		120 (85.71)	118 (84.89)	
Female	13 (9.29)		12 (8.63)	0.849	20 (14.29)	21 (15.11)	0.846
Smoker							
Ever	96 (69.06)		68 (49.28)		102 (73.38)	73 (52.90)	
Never	43 (30.94)		70 (50.72)	0.001	37 (26.62)	65 (47.10)	4.09 × 10^–4^

Significant *P*-values in bold font.

^*^Pearson’s chi-square test or the Fisher exact test for categorical variables and Student’s *t*-test for continuous variables. All *P-*values are two sided.

^**^Pack year = (packs per day) × (years smoked).

### Individual association of serum miRNAs with NMIBC risk

**Figure 1:**
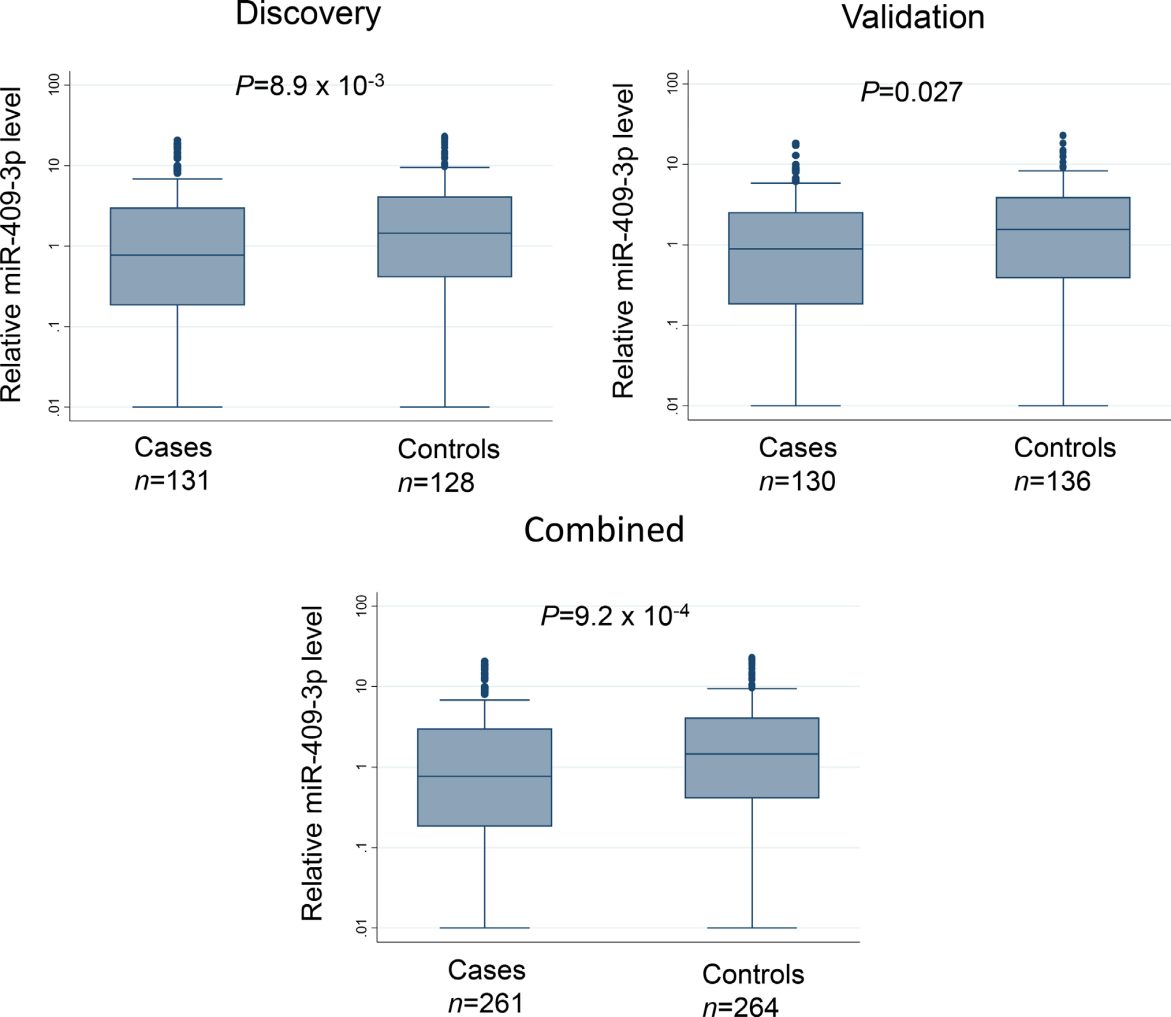
Box plots depicting relative serum expression level of miR-409-3p in NMIBC cases and controls in the discovery, validation, and combined sets Line inside the box represents median value and the box denotes values from 25th to 75th percentile. Upper and lower bars represent 95% confidence interval. *P*-values were determined by Wilcoxon rank sum test.

The 89 candidate miRNAs were assessed for serum expression using Fluidigm 96.96 Dynamic Array, a high throughput microfluidic array that enables simultaneous quantification by real-time PCR for 96 individual samples against 96 different miRNAs in a single experiment. After quality control and data cleaning (as described in Materials and Methods), the expression levels of 12 miRNAs were significantly associated with risk of NMIBC in the discovery set using tertiles of the miRNA levels in controls as cutoff points. When the same cutoffs were applied to the validation set, one miRNA, miR-409-3p, remained significantly associated with NMIBC with a significant trend for increased risk in the tertiles with reduced expression for both discovery set (*P* for trend = 0.013) and validation set (*P* for trend = 0.019) (Table 2). Pooled analysis indicated that this risk was the highest in the lowest tertile group (adjusted odds ratio [OR] = 2.21; 95% confidence interval [CI] = 1.40–3.50; *P* = 7.19 × 10^−4^). Box plots showing the differential serum levels of miR-409-3p in cases and controls in the discovery, validation, and combined sets is shown in Figure 1.

**Table 2:** Association of serum level of miR-409-3p with NMIBC risk

Level	Cases, *N* (%)	Controls, *N* (%)	OR^*^ (95% CI)	*P*-value	*P*_heterogeneity_
Discovery					
>2.4	38 (44.19)	48 (55.81)	1 (reference)		
0.46-2.4	39 (45.35)	47 (54.65)	1.09 (0.58–2.05)	0.785	
≤0.46	54 (62.07)	33 (37.93)	2.32 (1.20–4.49)	**0.012**	
*P* for trend				**0.013**	
Validation					
>2.4	36 (40.91)	52 (59.09)	1 (reference)		
0.46-2.4	40 (49.38)	41 (50.62)	1.46 (0.78–2.45)	0.238	
≤0.46	46 (58.97)	32 (41.03)	2.19 (1.14–4.22)	**0.019**	
*P* for trend				**0.019**	
Combined					
>2.4	74 (42.29)	101 (57.71)	1 (reference)		
0.46-2.4	87 (54.04)	74 (45.96)	1.30 (0.84–2.03)	0.242	0.520
≤0.46	83 (58.87)	58 (41.13)	2.21 (1.40–3.50)	**7.19**×**10**^**–4**^	0.905
*P* for trend				**7.22**×**10**^**–4**^	

Significant *P*-values in bold font.

^*^Adjusted by age, sex, smoking status and race.

### Individual association of serum miRNAs with onset of NMIBC and age of onset

Patients with early age onset of NMIBC may present distinct genetic and molecular features. To examine whether serum miRNAs may predict age of onset of NMIBC, we analyzed the 89 candidate miRNAs and their association with age of onset of NMIBC in both discovery and validation sets. Only two miRNAs, miR-409-3p and miR-342-3p, showed significant results in both groups. Patients with high level of both miRNAs displayed 1.8-fold (HR = 1.83, 95% CI, 1.41–2.38, *P* = 2.02 × 10^–5^) and 1.6-fold (HR = 1.60, 95% CI, 1.24–2.05, *P* = 2.05 × 10^–4^) increased risk of early onset of NMIBC in the combined population, respectively (Table 3). Patients with high serum level of these miRNAs have median age of onset of 63 years compared to 68 years for the low level group. Similar findings are found using the tertile analysis (data not shown). To check whether the association of miR-409-3p and miR-342-3p with onset of NMIBC is not confounded by association with age, we assessed the correlation of serum levels of these miRNAs with age of subjects separately in cases and controls. To have sufficient numbers, we combined the discovery and validation groups. We found serum levels of miR-409-3p and miR-342-3p were inversely correlated with age in both cases and controls (Spearman’s rho = –0.28 to –0.22, *P* < 0.001) (Supplementary Table 1).

**Table 3:** Association of serum miRNAs with age of onset of NMIBC

*Discovery*						*Validation*		*Combined*				
miRNA	Cases N (%)	HR^*^ (95% CI)	*P-*value	Median age of onset	Cases (%)	HR^*^ (95% CI)	*P-*value	Median age of onset	Controls N (%)	HR^*^ (95% CI)	*P-*value	Median age of onset
miR409-3p												
Low	69 (41.04)	1 (reference)		68	62 (45.07)	1 (reference)		69	131 (43.12)	1 (reference)		68
High	62 (57.73)	1.57 (1.10-2.25)	0.014	63	68 (58.49)	2.17 (1.47-3.21)	8.61 x 10^-5^	61	130 (58.13)	1.83 (1.41-2.38)	2.02 x 10^-5^	63
miR342-3p												
Low	55 (57.89)	1 (reference)		68	58 (61.70)	1 (reference)		68	136 (59.79)	1 (reference)		68
High	55 (45.08)	1.81 (1.26-2.60)	0.008	61	57 (42.86)	1.45 (1.02-2.06)	0.039	64	135 (43.92)	1.60 (1.24-2.05)	2.05 x 10^-4^	63

^*^Adjusted by age, sex and smoking status.

### Development of a predictive miRNA panel containing 3 serum miRNA ratios

Since there is no consensus on the standard method for normalization of data from circulating miRNA analysis, we also used the ratios between the expression values of all miRNAs as a quantification strategy, which has been identified as a robust and easily applicable method with potential for general application [14]. Each value of a single miRNA was compared with the values of all other remaining 52 miRNAs, and 1,378 ratios were obtained. A total of 147 miRNA ratios were significantly associated with risk of bladder cancer in the discovery set (Supplementary Table 2), and 6 of them remained significant in the validation set (Table 4). Among the latter group, half of the ratios were associated with decreased risk while the remaining ratios were associated with increased risk. The most significant protective effect between miRNA ratio and bladder cancer risk was observed in subjects with higher miR-409-3p/miR-423-5p ratio. These subjects displayed 50% lower risk for NMIBC (95% CI 0.34-0.74) compared to those with low ratio. On the other hand, the most significant increased risk was found in subjects with high miR-29c-3p/miR-331-3p ratio, who showed 2-fold increased risk of NMIBC (95% CI 1.34-3.03).

**Table 4:** Association of serum miRNA ratios with risk of NMIBC

	*Discovery*				*Validation*				*Combined*			
miRNA ratio	Cases *N* (%)	Controls *N* (%)	OR^*^ (95% CI)	*P-*value	Cases *N* (%)	Controls *N* (%)	OR^*^ (95% CI)	*P-*value	Cases *N* (%)	Controls (%)	OR^*^ (95% CI)	*P-*value
409-3p/423-5p												
Low	79 (58.96)	55 (41.04)	1 (reference)		78 (54.93)	64 (45.07)	1 (reference)		157 (56.88)	119 (43.12)	1 (reference)	
High	41 (42.27)	56 (57.73)	0.48 (0.27–0.84)	0.01	44 (41.51)	62 (58.49)	0.54 (0.31–0.94)	0.03	85 (41.87)	118 (58.13)	0.50 (0.34–0.74)	<0.001
29c-3p/331-3p												
Low	40 (42.11)	55 (57.89)	1 (reference)		36 (38.30)	58 (61.70)	1 (reference)		76 (40.21)	113 (59.79)	1 (reference)	
High	67 (54.92)	55 (45.08)	1.80 (1.00–3.22)	0.048	76 (57.14)	57 (42.86)	2.34 (1.31–4.16)	0.004	143 (56.08)	112 (43.92)	2.02 (1.34–3.03)	<0.001
331-3p/423-5p												
Low	70 (59.32)	48 (40.68)	1 (reference)		77 (53.47)	67 (46.53)	1 (reference)		147 (56.11)	115 (43.89)	1 (reference)	
High	43 (42.57)	58 (57.43)	0.43 (0.24–0.77)	0.004	41 (41.84)	57 (58.16)	0.55 (0.31–0.97)	0.039	84 (42.21)	115 (57.79)	0.51 (0.34–0.75)	<0.001
29a-3p/222-3p												
Low	43 (40.95)	62 (59.05)	1 (reference)		43 (41.75)	60 (58.25)	1 (reference)		86 (41.35)	122 (58.65)	1 (reference)	
High	78 (59.09)	54 (40.91)	1.97 (1.13–3.42)	0.016	84 (55.63)	67 (44.37)	1.85 (1.09–3.14)	0.023	162 (57.24)	121 (42.76)	1.90 (1.30–2.78)	0.001
146a-5p/423-5p												
Low	71 (57.26)	53 (42.74)	1 (reference)		79 (54.48)	66 (45.52)	1 (reference)		150 (55.76)	119 (44.24)	1 (reference)	
High	47 (43.93)	60 (56.07)	0.53 (0.30–0.92)	0.025	47 (44.34)	59 (55.66)	0.54 (0.31–0.94)	0.029	94 (44.13)	119 (55.87)	0.54 (0.37–0.79)	0.002
150-5p/331-3p												
Low	36 (40.00)	54 (60.00)	1 (reference)		48 (40.68)	70 (59.32)	1 (reference)		84 (40.38)	124 (59.62)	1 (reference)	
High	82 (57.75)	60 (42.25)	1.94 (1.10–3.42)	0.022	72 (53.33)	63 (46.67)	1.72 (1.01–2.92)	0.044	154 (55.60)	123 (44.40)	1.82 (1.24–2.66)	0.002

^*^Adjusted by age, sex and smoking status.

To determine a signature showing the best risk prediction, the combined effects of these serum miRNA ratios were investigated by incorporating their dichotomized status into our basic logistic regression model which included age, gender and smoking status. After testing a total of 63 possible combinations of the miRNA ratios, a signature of 3 miRNA ratios (miR-29a-3p/miR-222-3p, miR-150-5p/miR-331-3p, miR-409-3p/miR-423-5p) was able to improve the sensitivity and specificity for prediction of NMIBC risk over baseline model using epidemiologic variables of age, gender and smoking status in the combined set of subjects. A risk score based on the 3 miRNA ratio signature was calculated for all subjects. Patients with high-risk scores exhibited significantly increased risk of bladder cancer compared to those with low-risk scores (Discovery phase: OR = 3.53, 95% CI: 1.59–7.83, *P* = 1.89 × 10^–3^; validation phase: OR = 3.47, 95% CI: 1.68–7.19, *P* = 7.86 × 10^–4^; combined: OR = 3.52, 95% CI: 2.06–6.00, *P* = 3.78 × 10^–6^) (Table 5).

**Table 5:** Association of serum 3-miRNA ratio panel risk score with NMIBC risk

Sample set	Risk score	Cases, *N* (%)	Controls, *N* (%)	OR^*^ (95% CI)	*P-*value
Discovery	Low	16 (28.57)	40 (71.43)	1 (reference)	
	Medium	47 (58.75)	33 (41.25)	3.9 (1.81–8.40)	5.12 × 10^–4^
	High	37 (58.73)	26 (41.27)	3.53 (1.59–7.83)	1.89 × 10^–3^
	Trend				2.82 × 10^–3^
Validation	Low	21 (31.82)	45 (68.18)	1 (reference)	
	Medium	40 (49.38)	41 (50.62)	2.25 (1.10–4.61)	0.027
	High	46 (58.97)	32 (41.03)	3.47 (1.68–7.19)	7.86 × 10^–4^
	Trend				8.71 × 10^–4^
Combined	Low	37 (30.33)	85 (69.67)	1 (reference)	
	Medium	87 (54.04)	74 (45.96)	2.92 (1.74–4.90)	4.78 × 10^–5^
	High	83 (58.87)	58 (41.13)	3.52 (2.06–6.00)	3.78 × 10^–6^
	Trend				6.49 × 10^–6^

Ratio panel consists of miR-29a-3p/miR-222-3p, miR-150-5p/miR-331-3p, and miR-409-3p/miR-423-5p.

^*^Adjusted by age, sex and smoking status.

### Association of serum 3-miRNA ratio panel with epidemiological factors in NMIBC risk

To examine whether the effects of 3-miRNA ratio panel were modified by epidemiological factors, we combined the discovery and validation set data and performed stratified analyses based on age and smoking status. As shown in Supplementary Table 3, consistent and significant association of high risk scores with NMIBC risk was observed regardless of age dichotomized at 65 years (≤65 years: OR = 2.37, 95% CI: 1.30–4.31, *P* = 4.74 × 10^−3^; >65 years: OR = 2.66; 95% CI = 1.51–4.72, *P* = 7.65 × 10^−4^) and smoking status (ever smokers: OR = 3.45; 95% CI: 1.82–6.54; *P* = 7.02 × 10^-9^; never smokers: OR = 2.90; 95% CI: 1.45-5.81; *P* = 2.60 × 10^-3^). These results further supported the possibility that our miRNA ratio panel was not simply a surrogate for known risk factors, but a novel biomarker for unexplained variance in NMIBC risk.

### Pathway enrichment analysis for serum miRNA signature

**Figure 2:**
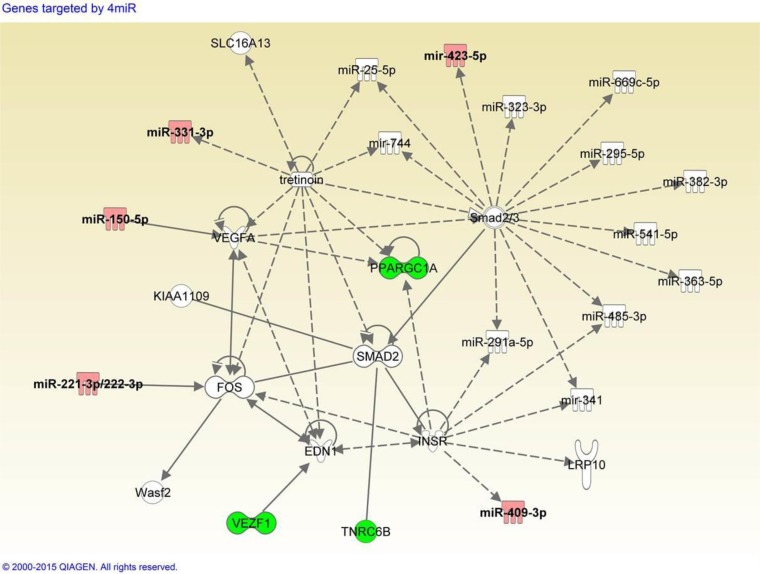
Integrative network analysis of 3-miRNA ratio combination panel and putative target genes Panel consists of miR-29a-3p/miR-222-3p, miR-150-5p/miR-331-3p, and miR-409-3p/miR-423-5p. Red: microRNAs listed in the 3-miRNA ratio combination panel. Green: genes targeted by more than four miRNAs in the combination panel.

To explore the possible biological pathways in which the serum miRNAs might be involved, pathway enrichment analysis was performed as described in Materials and Methods. Target identification from miRsystem reported 1798 genes, and 245 genes showed observed-to-expected ratio greater than 1.5 suggesting less likelihood for chance finding. Among these 245 genes, we identified five genes (*VEZF1, NFAT5, NFIX, PPARGC1A,* and *TNRC6B*), each of which was targeted by four miRNAs in the 3-miRNA ratio panel. A custom dataset was created with these five target genes and six miRNAs in IPA. Core analysis was performed on the custom dataset, and we found one network containing three genes (*VEZF1, PPARGC1A*, *TNRC6B*) and five miRNAs (miR-150-5p, miR-222-3p, miR-331-3p, hsa-miR-409-3p, miR-423-5p) from our custom dataset. We filtered out genes from the network, which were only linked to ubiquitin C gene, a universal degradation mechanism for multiple proteins. As shown in Figure 2, several miRNAs and their targets were linked to retinoid acid receptor (RAR) activation which involves *FOS*, *PPARGC1A*, *SMAD2*, *VEGFA* and tretinoin. Tretinoin is a retinoid suggested to possess chemopreventive activity via RAR signaling.

## DISCUSSION

In this study, we have focused on the role of miRNAs as potential diagnostic/risk prediction biomarkers of bladder cancer by conducting global and targeted miRNA profiling of serum samples. We discovered one miRNA miR-409-3p and six cancer-related miRNA ratios (miR-29a-3p/miR-222-3p, miR-29c-3p/miR-331-3p, miR-146a-5p/miR-423-5p, miR-150-5p/miR-331-3p, miR-331-3p/miR-423-5p and miR-409-3p/miR-423-5p) were significantly associated with NMIBC risk in both discovery and validation groups. In addition miR-409-3p and miR-342-3p were associated with early onset of NMIBC suggesting their roles in early cancer development. These miRNAs were highly detectable in sera from bladder cancer patients pointing to their utility as noninvasive markers. We also developed a risk score by combining 3-miRNA ratios to distinguish NMIBC cases from controls. Once confirmed in independent studies, the miRNA panel may be applied to improve the discriminatory performance of bladder cancer risk prediction model incorporating host, clinical, and epidemiologic characteristics.

Although previous studies have reported serum miRNA expression profiles predicting cancer risk or outcome in several solid tumors [10, 11, 14–16], studies focusing on the association of serum miRNA expression with bladder cancer were rather limited. Based on the qRT-PCR analysis of 22 selected miRNA candidates in 148 patients with NMIBC or muscle invasive bladder cancer and 115 control subjects, Scheffer AR *et al.* [17] failed to demonstrate a significant difference in serum miRNA levels between cases and controls. Because of the limited number of candidate miRNAs, small sample size and low detection rate of some miRNAs, it is possible that this study did not have sufficient statistical power to identify diagnostic biomarkers for bladder cancer. Another study was carried out in 44 cases across all stages and 34 controls in European population to validate 11 miRNAs associated with bladder cancer. The results suggested that miR-141 discriminates patients with bladder cancer with 70.5% sensitivity and 73.5% specificity [18]. In one recent publication, serum samples from Chinese subjects were screened with next-generation sequencing in 10 cases and 10 controls to identify differentially-expressed miRNAs. A total of 26 miRNAs were found significant and was used in subsequent training and validation profiling in 240 and 220 samples, respectively. Six miRNAs (miR-152, miR-148b-3p, miR-3187-3p, miR-15b-5p, miR-27a-3p and miR-30a-5p) were reported to be able to differentiate bladder cancer patients with an AUC of 0.899 [13]. In our study, we also detected and profiled miR-152, miR-148b, and miR-27a-3p; however, the results were not significant. To the best of our knowledge, this multi-phase study is the first to combine global and targeted miRNA profiling using Taqman miRNA arrays and Fluidigm’s multiplex, high-throughput platform to analyze serum miRNAs associated with NMIBC in a Caucasian population.

In this study, we identified potential role of serum miR-409-3p, miR-342-3p, and a 3-miRNA ratio panel (miR-29a-3p/miR-222-3p, miR-150-5p/miR-331-3p, miR-409-3p/miR-423-5p) as risk predictors in NMIBC. The biological significance and association of these miRNAs with cancer have been previously reported. In particular, three miRNAs have been confirmed to be dysregulated in bladder cancer. MiR-409-3p was reported to inhibit migration and invasion of bladder cancer cells by targeting c-Met [19]. MiR-150-5p functions as a tumor promoter in reducing chemosensitivity and promoting invasiveness of muscle-invasive bladder cancer cells lines by targeting PDCD4 [20], while miR-222-3p overexpression in bladder cancer tissues is associated with poor prognosis possibly via suppression of p27^Kip1^ [21]. The other three miRNAs, although not implicated in bladder cancer, have been shown to be associated with other types of cancers. Several targets have been biologically proven and linked to the roles of miR-29a-3p in various cancers. Indirect downregulation of matrix metalloproteinase 2 (MMP2) and E-cadherin by miR-29a-3p through Krüppel-like transcription factors 4 (KLF4) was reported in colon cancer cells [22]. Interestingly, KLF4 inhibition has been shown to promote invasion and migration *in vitro* [23] and linked to poor progression and early recurrence [24] in bladder cancer. In hepatocellular carcinoma, miR-29a-3p suppressed cell growth by inhibition of Secreted protein acidic and rich in cysteine (SPARC) [25] while SPARC-deficient mice were shown to develop urothelial carcinoma much earlier than mice expressing SPARC [26]. By targeting Lamin γ2 (LAMC2), miR-29a-3p was reported to suppress migration and invasion in head and neck cancers [27], and overexpression of LAMC2 was found to enhance invasiveness *in vivo* [28]. In glioblastoma cells, miR-331-3p suppresses neuropilin-2 (NRP-2) expression directly [29], and NRP-2 has been reported to predict treatment response of bladder cancer [30]. MiR-331-3p has been shown to suppress ERBB2 signaling in prostate cancer [31] while ERBB-2 was indicated to be correlated with chemoradiation therapy resistance in muscle invasive bladder cancer patients [32]. In addition, E2F1 was a confirmed direct target of miR-331-3p in gastric cancer [33], and expression of *E2F1* and other associated genes were able to predict invasive progression of bladder cancer [34]. MiR-342 is a potential tumor suppressor and is known to be downregulated in several cancer types. For example, downregulation of miR-342 has been associated with tamoxifen resistant tumors [35]. MiR-342 was shown to be a negative regulator of E2F1 affecting MYC expression in lung cancer cells [36] and also negatively regulate FOXM1 and FOXQ1 expression in colorectal cancer cells [37]. Finally, miR-423-5p has been reported to be downregulated in gastric cancer [38] and a biomarker for colorectal cancer diagnosis [39]. Our results here provided evidence suggesting that these miRNAs might be involved in carcinogenesis of non-muscle invasive bladder cancer. Further study is required to clarify the role of these miRNAs in bladder cancer development.

Interestingly, we found the serum levels of miR-409-3p and miR-342-3p inversely correlated with age, which contributed to its association with early onset of bladder cancer. Cases with higher expression of these miRNAs had earlier diagnosis of NMIBC compared to lower level group (by 5 years) even though the two miRNAs were protective in terms of risk. It should be noted the cases and controls were frequency-matched in terms of age and gender and that the median cutoff of serum miRNA level in the cases for age of onset analysis is lower than that of the case-control analysis using median cutoff for controls. Previous studies have implicated miR-409-3p to be involved in immune-regulatory function in natural killer and T cells [40, 41]. It is possible that decreasing serum levels of this miRNA by age was due to decreased immune function during aging. More in depth mechanistic studies are needed to characterize the association. The relatively large sample size allowed us to examine how our miRNA panels performed in various age and smoking status strata. The elevated risks were consistent between age groups and when stratified by smoking status. Interaction between miRNA panel and age or miRNA panel and smoking was not significant (*P* > 0.05) suggesting independent association with NMIBC risk.

Delineation of the molecular pathways involved in bladder oncogenesis may expand the quest for much-needed diagnostic biomarker discovery [42]. MiRNA target prediction tools coupled with the network analysis enabled us to determine pathways targeted by miRNA panels. In the network composed of our panel miRNAs and target genes, we found the miRNA panel could modulate the activation of RAR pathway involving retinoids. Retinoids are metabolites of vitamin A (retinol), and have been shown to bind the heterodimers of retinoic acid receptors (RARs) and/or retinoid X receptors (RXRs). The heterodimer of RAR and RXR binds to retinoic acid response elements (RAREs) and regulate downstream transcription via interaction with other coactivator and corepressors. Upon conjugation of retinoids, RAR/RXR dimers recruit more coactivators such as histone deacetyltransferases which are correlated with transcriptional activation of RAR target genes. Therefore, retinoic acid has been considered to be responsible for the activities of vitamin A [43]. Retinoids were found to inhibit cell cycle by upregulation of cell-cycle inhibitors [44] and degradation of cyclin D1 [45]. They can also trigger apoptosis via induction of TNF-related apoptosis-inducing ligand (TRAIL) signaling [46]. The epidemiological association between retinoids and bladder cancer were found back in 1979 [47]. Subsequent studies showed that dietary supplement containing a retinoid (all-trans-N-(4-hydroxyphenyl) retinamide (4-HPR)) was able to decrease the depth of tumor invasion in a chemically-induced bladder cancer mouse model [48]. In 1985, de Bolla *et al.*, reported that retinoid acid receptor expression was negatively correlated with recurrence [49]. The mechanisms behind these observations have not been fully clarified in bladder cancer, but transrepression of activator protein-1 (AP-1) which was composed of FOS and JUN by ligand-bound RAR/RXR complex has been suggested to account for the biological activity of retinoids in a mouse fibroblast cell line [50]. Inhibition of FOS has been reported to impact VEGFA protein level [51] and the downregulation of VEGFA could be linked to reduced PPARGC1A (PGC-1A) expression [52]. Based on prediction of target genes in miRsystem, miR-222-3p inhibits FOS, miR-150-5p inhibits VEGFA, and PPARGC1A is targeted by miR-150-5p, miR-409-3p and miR-222-3p. The involvement of our miRNA panels along the FOS-VEGFA-PPARGC1A axis suggests that the miRNAs might either serve as a proxy for the activation of RAR pathway, or a modulator of cellular response to retinoids. Besides miR-150 and miR-222, altered expression of these miRNAs in NMIBC tumor tissues have not been reported in literature [53]. Potential of these miRNA panels as a biomarker for tumor susceptibility warrants further investigation. In addition, VEGFA and INSR proteins in the above network have been under active investigation for targeted therapy [54]. Besides risk, the possibility of our miRNA panel to predict treatment response may warrant investigation to help refine personalized medicine.

Our study has several strengths including the multi-stage design with initial global screening of stably-expressed circulating miRNAs followed by targeted assessment in discovery and validation phases. Compared to most other studies of circulating miRNAs as risk predictors, the study is sufficiently powered for stratified analyses by covariate risk factors such as age and smoking status in order to detect biomarker interactions. We collected comprehensive epidemiologic and clinical information which are amenable for more integrative analysis. However, there are also some limitations including the relatively small sample size in one racial/ethnic population to prevent broad generation, and the retrospective study design that allows the possibility for reverse causation. Additionally, the normalization of circulating miRNA expression and selection of internal controls are still challenging and ongoing research. Further studies are needed to determine the optimal reference miRNAs for qPCR analysis. Although we have maintained consistent quality control of our blood samples, variation in sample storage time and subject health conditions before blood collection might affect the measurement results. Nevertheless, some of these confounders would more likely bias towards the null.

In conclusion, using miRNA array profiling combined with Fluidigm platform, we provided evidence that serum miRNAs could potentially serve as non-invasive biomarkers for NMIBC. Further replication in independent prospective studies is warranted. The serum 3-miRNA ratio panel may contribute toward clinical application for noninvasive diagnosis, early detection or risk prediction of bladder cancer. Enrichment of target genes linked to RAR signaling provides biological plausibility regarding bladder cancer development and identifies novel candidates for therapeutic intervention.

## MATERIALS AND METHODS

### Study population and epidemiological data

This study included a total of 290 NMIBC cases and 288 healthy controls. Cases were recruited from The University of Texas MD Anderson Cancer Center and Baylor College of Medicine as a part of an ongoing bladder cancer case control study since 1999. As described previously [55], cases were all newly diagnosed, histologically confirmed, and previously untreated with chemotherapy or radiotherapy at the time of recruitment. Controls were healthy individuals without prior cancer history (except for non-melanoma skin cancer) and were recruited through Kelsey-Seybold Clinics, the largest multispecialty physician group in the Houston metropolitan area. Controls were frequency matched to cases on age (±5 years), sex and ethnicity. To control for population stratification, only Caucasians were included in this study as more than 90% of our recruited participants belonged to this group. All participants provided written informed consent before the collection of epidemiological data and blood samples.

Epidemiological data, including demographics, family history and smoking status, were collected by MD Anderson interviewers during a 45 min interview. Smoking status was classified as never-smokers (never smoked or smoked < 100 cigarettes in their lifetime) and ever smokers (smoked ≥ 100 cigarettes in their lifetime). Immediately after each interview, a 40 ml peripheral blood sample was collected, of which about 10 ml was drawn into a gold top serum-separating tube and processed for serum extraction within two hours. Extracted serum samples were then transferred to liquid nitrogen storage tanks until needed. All of the human study participation procedures were approved by the Institutional Review Boards at the University of Texas MD Anderson Cancer Center, the Baylor College of Medicine, and the Kelsey-Seybold Clinics.

### RNA isolation

Isolation of total RNA from serum was carried out using miRNeasy Mini Kit (QIAGEN, Valencia, CA, USA) following the manufacturer’s protocol. For each miRNA profiling and real-time PCR assay, 700 μL serum was used. Single-stranded synthetic miRNA (*C. elegans,* cel-miR-39) was spiked into serum as a control for evaluation of successful extraction. RNAs were eluted twice with 30 μL of water and stored at –80° C until ready for use. RNA concentration was measured by NanoDrop ND-1000 spectrophotometer (Thermo Scientific, DE, USA). The RNA concentrations for all samples ranged from 42 ng/μl to 112 ng/μl (mean = 80.4 ± 17 ng/μl). To ensure sufficient sample quality, the amount of serum and spike-in miRNAs prepared from the same batch were carefully kept consistent throughout all experiments. The effect of hemolysis during the serum preparation procedure [56] has been checked by detecting serum free hemoglobin level in the VITROS^®^ Fusion 5.1 Chemistry System (Ortho Clinical Diagnostics) at the University of Texas MD Anderson Cancer Center Core Laboratory. All serum samples used for further analysis were free from significant hemolysis.

### MiRNA array profiling

Global miRNA profiling was performed on serum samples from ten NMIBC patients and ten sex- and age-matched healthy subjects using TaqMan^®^ Array Human MicroRNA Card Set v3.0 (Applied Biosystems, Foster City, CA, USA), which contains probes for 754 human miRNAs. Briefly, total circulating RNA was reverse-transcribed using TaqMan^®^ MicroRNA Reverse Transcription Kit and the Megaplex™ RT Primers followed by a pre-amplification reaction using TaqMan^®^ PreAmp Master Mix and Megaplex PreAmp Primers. Preamplified target cDNAs were then mixed with TaqMan^®^ Universal PCR Master Mix and loaded onto the TaqMan^®^ MicroRNA Array. Quantitative real-time PCR was performed on the 7900HT Fast Real-Time PCR System (Applied Biosystems). Expression threshold for each miRNA detector was automatically determined. After initial screening, a total of 166 miRNAs could be detected. Among these, we selected 89 miRNAs that are related to cancer pathways or reported in bladder cancer based on literature [57–62] for further testing in discovery and validation sets (Supplementary Table 4). MiRNAs with a Ct value < 35 and a missing rate < 25% in both study groups were considered as stably detectable candidates for further analysis.

### MiRNA expression by Fluidigm microfluidics dynamic arrays

Selected miRNAs were measured using high-throughput BioMark™ HD Real-Time PCR system (Fluidigm, San Francisco, CA, USA). Reverse transcription and pre-amplification reaction were carried out using the same protocol as shown in miRNA array profiling, except primers for two spike-in miRNAs (cel-miR-39 and cel-miR-54) were added. PCR Products were cleaned up using an enzymatic digestion approach by Exonuclease I (NEB, #M0293L) to remove primers. After pre-amplification, a 5 μL sample mixture was preparedcontaining 1 × TaqMan Universal Master Mix (No UNG), 1 × DNA sample Loading Reagent (Fluidigm) and each of pre-amplified cDNA. 5 μL of assay mix was prepared with 1 × each of TaqMan miRNA assay and 1 × Assay Loading Reagent (Fluidigm). The IFC controller HX (Fluidigm) was used to distribute the sample mix and assay mix from the loading inlets into the 96.96 Dynamic array reaction chambers. After loading, the chip was placed in the BioMark Instrument for real-time PCR at 95° C for 10 min, followed by 40 cycles at 95° C for 15 sec and 60° C for 1 min. Data was analyzed with Real-Time PCR Analysis Software in the BIOMARK instrument (Fluidigm). Each PCR reaction was done in duplicate, including blank control, negative control and one calibrator DNA. The assay for cel-miR-39 was used for evaluation of RNA quality and for normalization in individual miRNA analysis; the assay for cel-miR-54 was used as negative control, and a calibrator DNA consisting of 10 random serum samples was used for comparison of results of different independent plate assays. Data that met one of the following criteria were excluded from further analysis: 1) the Ct value of duplicate assays differ from each for more than one cycle; 2) samples with a Ct value > 25 or < 16 in spike-in miRNAs; 3) miRNAs with a missing rate > 25%; 4) data points outside 5 standard deviations were considered outliers and excluded from analysis. After data cleaning, 53 out of the 89 miRNA candidates were subjected to further analyses. For each miRNA obtained after data cleaning, the Ct value was normalized to the average expression of cel-miR-39 and then transformed to the corresponding expression value using the 2^-ΔΔCt^ method. To generate miRNA ratios, the normalized mean Ct values were subjected to the 2^-ΔΔCt^ method, and then one expression ratio was calculated for each pair of miRNAs.

### Pathway enrichment analysis

Serum miRNA panels found to be significantly associated with bladder cancer were further analyzed by pathway enrichment analyses. We first identified genes targeted by the miRNA panel using miRsystem [63] which integrates results from seven prediction algorithms including DIANA [64], miRanda [65–67], mirBridge [68], PicTar [69], PITA [70], rna22 [71], and TargetScan [72–74], and two experimentally validated databases, TarBase [75] and miRecords [76]. Network enrichment was performed using QIAGEN’s Ingenuity^®^ Pathway Analysis (IPA^®^, QIAGEN Redwood City, CA, USA; www.qiagen.com/ingenuity). Selected miRNAs and their target genes were entered to create a custom dataset for IPA core analysis. Networks enriched with most miRNAs and target genes were subjected to visual inspection. We removed the ubiquitin C (UBC)-related elements because UBC conjugation occurs commonly in the degradation of a broad spectrum of proteins without known significance in bladder cancer.

### Statistical analysis

Statistical analyses were performed using Intercooled STATA version 10 (StataCorp, College Station, TX, USA). Pearson chi-square test (or the Fisher exact test, for categorical variables, sex, race and smoking status) or Student’s *t*-test (for continuous variables, age) was used to evaluate differences in patient characteristics. Wilcoxon rank sum test was used to compare serum level of miRNAs in cases and controls. For analysis of individual miRNAs and their association with NMIBC risk, median and tertile levels of miRNAs in controls in the combined groups was used to categorize cases and controls. Odds ratios (ORs) and 95% confidence intervals (CIs) were estimated using unconditional multivariable logistic regression analysis, adjusting for possible confounding by age, sex and smoking status. For analysis of individual miRNA association with age of onset of NMIBC, the median level in cases in the combined group was used to dichotomize miRNA level in the discovery and validation sets. Cox proportional hazard model adjusted for covariates was used to estimate association of miRNA level with age of onset. The ratios between two miRNA serum levels were generated using all possible combinations. The median miRNA ratio in the combined group was used to dichotomize miRNA ratios in the discovery and validation phases. Logistic regression was performed to determine the significance of each miRNA ratio as a predictor of NMIBC risk. Combinations of significant miRNA ratios were plugged into a multiple logistic regression model which included age, gender and smoking status. A single risk score comprised of the weighted sum for the 3 miRNA ratios were calculated and the weight is the adjusted log (OR) representing the estimated relative risk in log scale. The risk scores were dichotomized by the median of risk scores in controls of discovery phase. All *P*-values are two-sided, and *P-*value ≤ 0.05 was considered statistically significant.

## SUPPLEMENTARY MATERIALS TABLES







## Abbreviations

NMIBC: non-muscle invasive bladder cancer
miRNA: microRNA
