# Supplementary material for: Serum microRNAs as predictors of risk for non-muscle invasive bladder cancer
Source: Oncotarget. 2018 Feb 12;9(19):14895–908. doi: 10.18632/oncotarget.24473 (PMC5871085; doi:10.18632/oncotarget.24473)
Supplement: Supplementary file 1 [file oncotarget-09-14895-s001.pdf]

Serum microRNAs as predictors of risk for non-muscle invasive bladder cancer

SUPPLEMENTARY MATERIALS

Supplementary Table 1: Correlation of serum levels of miR-409-3p and miR-342-3p with age in NMIBC cases and controls

	N[*]	Spearman's rho	*P*-value
miR-409-3p			
Cases	261	–0.2309	0.0002
Controls	264	–0.2841	<0.0001
miR-342-3p			
Cases	271	–0.2831	<0.0001
Controls	258	–0.2156	0.0005

[*]Numbers are less than total due to some subjects with undetectable level

Supplementary Table 2: Significant miRNA ratios in discovery phase. See Supplementary_Table_2

Supplementary Table 3: Stratified analysis of the 3-miRNA ratio panel by age and smoking status in the combined group

Risk score of Stratified analyses	Cases, *N* (%)	Controls, *N* (%)	OR* (95% CI)	*P*-value
Age ≤ 65				
Low	31 (14.98)	56 (25.81)	1 (reference)	
High	66 (31.88)	45 (20.74)	2.37 (1.30–4.31)	4.74×10^{-3}
Age > 65				
Low	20 (9.66)	57 (26.27)	0.54 (0.27–1.09)	0.087
High	90 (43.48)	59 (27.19)	2.66 (1.51–4.72)	7.65×10^{-4}
Never smokers				
Low	17 (8.29)	52 (24.07)	1 (reference)	
High	44 (21.46)	50 (23.15)	2.90 (1.45–5.81)	2.60×10^{-3}
Ever smokers				
Low	33 (16.10)	61 (28.24)	1.77 (0.87–3.59)	0.112
High	80 (66.12)	41 (33.88)	3.45 (1.82–6.54)	7.02×10^{-9}

*Adjusted by age, sex and smoking status when appropriate.

Supplementary Table 4: Missing rate of 89 serum detectable miRNAs. See Supplementary_Table_4
